# Luminescent photonic crystals with multi-functionality and tunability†
†Electronic supplementary information (ESI) available: Control experiments and the corresponding emission and reflection spectra. See DOI: 10.1039/c6sc01703g


**DOI:** 10.1039/c6sc01703g

**Published:** 2016-05-25

**Authors:** Hong Wang, Xinggui Gu, Rongrong Hu, Jacky W. Y. Lam, Deqing Zhang, Ben Zhong Tang

**Affiliations:** a HKUST-Shenzhen Research Institute , No. 9 Yuexing 1st RD, South Area, Hi-tech Park, Nanshan , Shenzhen 518057 , China . Email: tangbenz@ust.hk; b Department of Chemistry , Hong Kong Branch of Chinese National Engineering Research Center for Tissue Restoration & Reconstruction , Institute for Advanced Study , Institute of Molecular Functional Materials , Division of Biomedical Engineering , Division of Life Science and State Key Laboratory of Molecular Neuroscience , The Hong Kong University of Science & Technology , Clear Water Bay , Kowloon , Hong Kong , China; c Guangdong Innovative Research Team , SCUT-HKUST Joint Research Laboratory , State Key Laboratory of Luminescent Materials and Devices , South China University of Technology , Guangzhou 510640 , China; d Beijing National Laboratory for Molecular Sciences , Key Laboratory of Organic Solids , Institute of Chemistry , Chinese Academy of Sciences , Beijing 100190 , PR China

## Abstract

The marriage of reflected light originating from photonic crystals (PCs) and emitted light would create miraculous phenomena. However, traditional luminophores cannot avoid the problem of aggregation-caused quenching. To solve this problem, we develop a general method to incorporate aggregation-induced emission luminogens (AIEgens) into PCs *via* physical absorption or chemical reaction. The resulting luminescent PCs display diverse structural colors in response to water stimulation, due to the swelling of the aqueous medium. Such a water-tunable photonic band gap red-shift has the ability to modulate the AIEgen emission, as well as narrowing its full width at half maximum (FWHM), which allows the luminescent PC to behave as a smart intramolecular filter that is capable of creating arbitrary light from only one material. In addition, the filter is believed to modulate the broad emission spectra of AIEgens arising from different conformations. Furthermore, the luminescent PC can respond to ethanol stimulation due to two factors: (a) swelling of the aqueous medium (external tuning); and (b) expansion of nanoparticles (internal tuning). By exploiting the synergy of the external-internal tuning, the emission wavelength and intensity can be finely changed. Both the water- and ethanol-tunable emission shift fit to a linear relationship, and thus the luminescent PC could be able to quantitatively detect humidity in the environment and alcohol in wine.

## Abstract 2

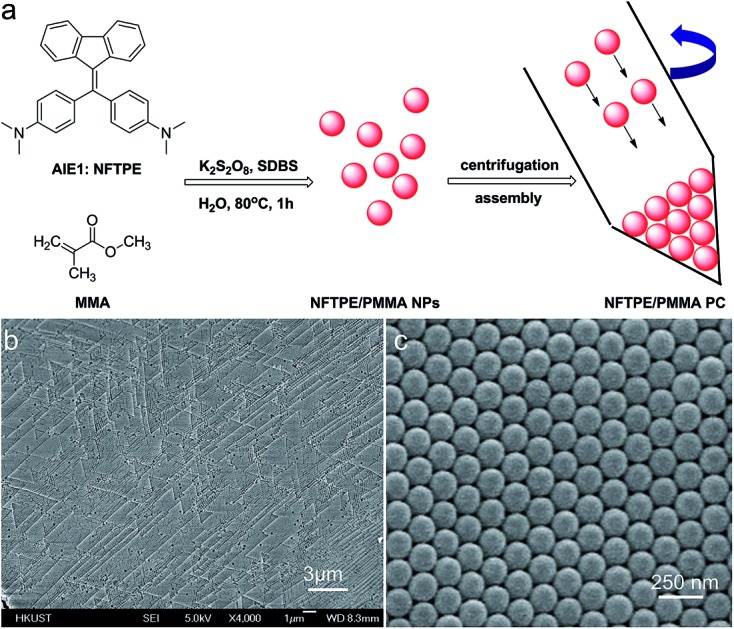
We develop a general method to incorporate aggregation-induced emission luminogens into photonic crystals (PCs) and the resulting luminescent PCs display diverse structural colors in response to water stimulation.

## Introduction

In biological systems, the effects of photonic crystals (PCs) have created unique and amazing structural colors, building a colorful world.^1^ Many living organisms can exhibit iridescent color or switch different colors by tuning their PC structures for social communication, sexual attraction, or environmental camouflage.^2^ For example, *Pentapodus paradiseus* can reversibly alter color from blue to red under different osmotic pressures;2*b**Tmesisternus isabellase* can change its structural color from gold to red in response to humidity variation;^3^*Paracheirodon innesi* can convert from cyan, in the normal state, to yellow, in the stressful state;2*a* and *Furcifer pardalis* can switch from green to orange upon excitation.^4^ Such structural color changes are critically dependent on external stimulation. One popular approach, energy-based stimuli, for example use photons, heating, pressure and chemicals. On the other hand, coloration can be achieved by exploiting water as a medium-active stimulus, because it is much easier to be obtained by living organisms. However, studies of water-active stimuli are very limited although there have been studies regarding animal evolution.^5^ Inspired by Nature, it is believed that such water-active color-tuning is of great excitement and would bring a lot of potential applications.

So far, research on artificial PCs has been focused on incorporating self-assembled PC structures into a stimuli-responsive matrix (*e.g.*, polymers or hydrogels).2c,^6^ In comparison to natural PCs, they are much less smart. A major problem is the single functionality depending on the periodic crystal packing. That is, only structural color changes can be realized by adjusting the packing pattern, which limits their applications in the field of multi-function materials. Thus it is of great interest to endow PCs with internal functional properties besides such structure-induced properties.

There are many approaches for tuning the structural colors of PCs. One is designed to change the lattice spacing through the application of external stimuli on the matrix.^7^ Typical examples are swelling/deswelling hydrogel/PC composites using water,^8^ or stretching/releasing PDMS/PC materials *via* mechanical stress.^9^ An alternative method is self-tuning the PCs (*i.e.*, internal tuning),^10^ such as swelling/shrinking the hydrophilic layer of the PS-*co*-PDMAA opal hydrogel.^11^ However, both these approaches are rarely applied in one system. Herein, we realized multi-dimensional tuning by exploiting the internal-external tuning synergy, where both the color and intensity are able to be modulated.

Photonic crystals are known for their ability to control the propagation of light *via* the photonic band gap (PBG), and displaying the emitted light.^12^ The question is whether miraculous phenomena would arise when both reflected and emitted light were combined together? However, traditional fluorophores are subjected to aggregation-caused quenching (ACQ), where their emissions are weakened in the solid state. Conversely, aggregation-induced emission luminogens (AIEgens) display excellent emission efficiencies in the solid state and have full color emissions.^13^ Thus, such AIEgens are the most promising materials for high emitting PCs.

We are interested in fabricating AIEgen-functionalized PC structures, which are promising materials for sensors. Recently, we reported the silole-infiltrated SiO_2_ inverse opal PC for detecting organic vapors (VOCs).^14^ The crystallization of AIEgens was transformed with a spontaneous color change of the PC upon encountering the VOCs. Another example is the use of a silole-based PC film as a fluorescent probe to selectively detect Hg^2+^ and Fe^3+^.^15^ However, multi-functional and multi-responsive luminescent PCs have so far not been demonstrated.

Here, we report a unique PC system composed of AIEgen-based poly(methyl methacrylate) (PMMA) nanoparticles (NPs), where the color of both the reflected and emitted light can be tuned simultaneously in response to water stimulation. This simple and controllable system allows the modulation of single- or multi-emission from only one material. Induced by the water-active stimulus, the PBG of the PC is bathochromically shifted, leading to a red-shift in emission with a narrow full width at half maximum (FWHM). We find that such a luminescent PC system could respond to both external (*e.g.*, swelling by water) and internal tuning (*e.g.*, swelling by ethanol), where the intensity and wavelength of emission are changed. Thus, it is believed that this system is a good dual-sensor for detecting humidity and alcohol.

## Experimental

### Materials

Methyl methacrylate (MMA), potassium persulfate (K_2_S_2_O_8_, ACS reagent, Sigma), sodium dodecyl benzene sulfonate (SDBS, technical grade, Sigma), and ethanol absolute (Merck) were used as received. AIEgens NFTPE (AIE1) and TPE-containing diacrylate (AIE2) were prepared following the literature procedures.^16^ THF was distilled under normal pressure from sodium benzophenone ketyl under nitrogen immediately prior to use.

### Characterization

SEM images were collected on a JEOL-7100F scanning electron microscope operating at an accelerating voltage of 20 kV. Photoluminescence (PL) spectra were recorded on a Perkin-Elmer LS 55 spectrofluorometer. The reflectance spectra were detected on a HR-4000CG-UV-NIR spectrometer.

### Synthesis of luminescent photonic crystals

The luminescent PCs were prepared *via* a modified emulsion polymerization following a literature method.^17^ In brief, MMA (3 mL, 47.7 mg) was dispersed into 25 mL water, which contained SDBS (5 mg). The mixture was heated to 80 °C with stirring. Then, the AIEgen in THF (2 mL, 2.5 mg mL^–1^) was dropwise added into the above solution. After adding K_2_S_2_O_8_ (0.06 g in 1.5 mL H_2_O), the polymerization was carried out at 80 °C for 1 h. The luminescent nanoparticles were centrifuged and washed with water three times. Finally, the luminescent PC hydrogels were obtained using centrifugation at 12 000 rpm for 15 min.

### The external tuning of PCs upon water-active stimulus

Both the band gap and emission peak of the AIEgen-based PCs were bathochromically shifted upon adding water. After centrifugation, such a PC hydrogel (50 μL) was added into a quartz cell. Then deionized water (0, 25, 50, 75, 100, 125, and 150 μL) was added into the cuvette. After sonication for 2 min, the reflectance and PL spectra were collected. The same procedure was used for the response to the stimulation of ethanol.

## Results and discussion

**Fig. 1:**
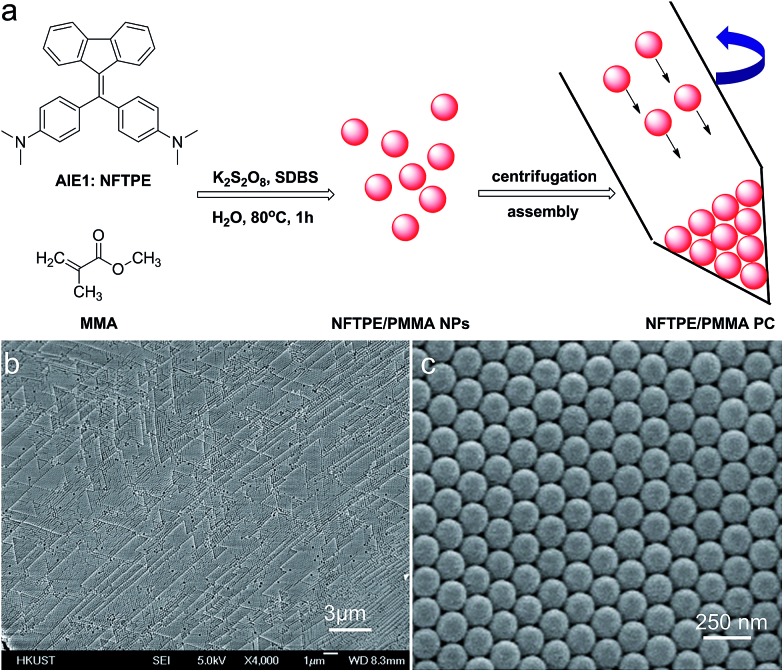
(a) Schematic illustrating the molecular structure of NFTPE AIE1 and the assembly process of the NFTPE/PMMA PC. (b and c) SEM image (b) and enlarged image (c) of NFTPE functionalized poly(methyl methacrylate) photonic crystals.

The AIEgen-loaded PMMA nanoparticles were synthesized *via* a modified emulsion polymerization.^17^ Specifically, AIE1 NFTPE (Fig. 1a), exhibiting yellow-green emission, was dissolved in THF solution, and mixed with hot MMA/SDBS aqueous solution, followed by addition of K_2_S_2_O_8_ as initiator. After 1 h incubation at 80 °C, the solution turned to a light creamy yellow, indicating MMA polymerization. Driven by hydrophobic interactions, NFTPE are encapsulated by PMMA NPs, with the hydrophilic ends of SDBS dissolved in water. The resulting luminescent NPs were washed with water through centrifugation. To induce the PC hydrogel assembly, the purified NPs were then centrifuged at high speed (12 000 rpm), illustrated in Fig. 1a, after which an opal structure was obtained. Fig. 1b and c show the scanning electron microscopy (SEM) images of the PC after drying, where the NPs with a uniform diameter of 120 nm are in a face-centered cubic arrangement. It is believed that the uniform size and centrifugation speed of the luminescent NPs play critical roles in making the high performance AIE/PMMA PC. The former is a prerequisite, and the latter is the determining factor. If the speed is lower than 12 000 rpm, the PC cannot form, while large aggregates would occur at higher centrifugation speeds (>12 000 rpm).

**Fig. 2:**
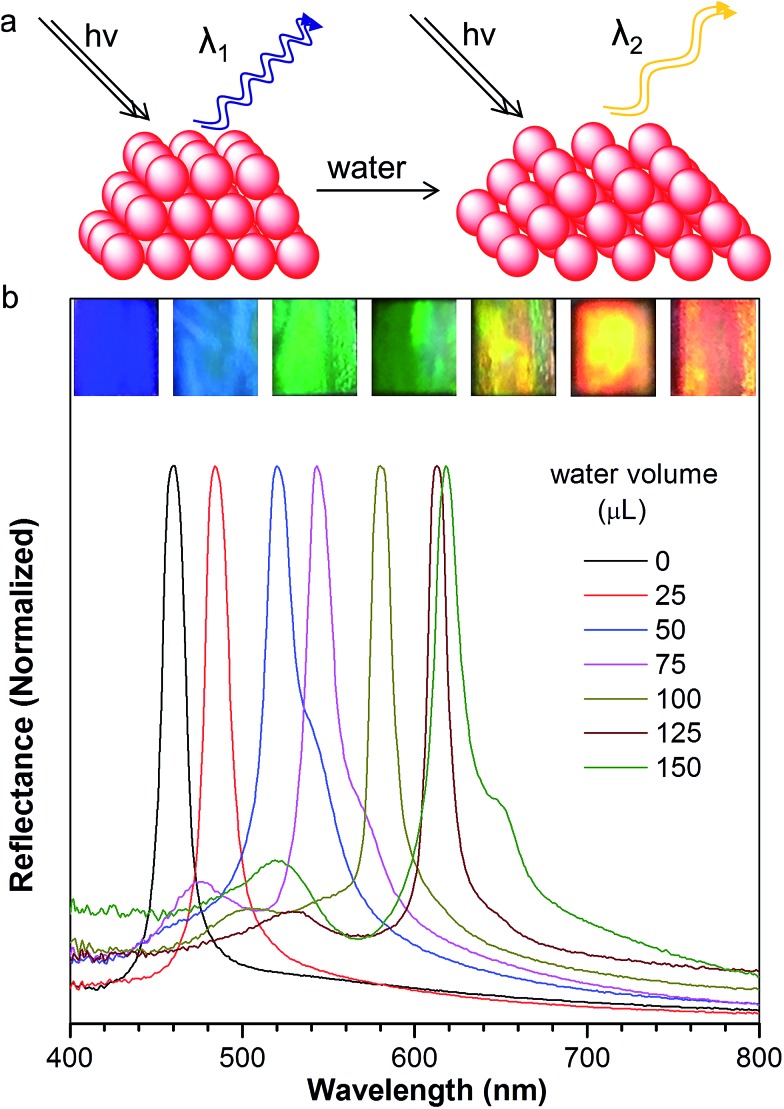
(a) Schematic illustrating the swelling process of the AIEgen-loaded photonic crystals. (b) Photographs (inset) and corresponding reflection spectra of the NFTPE/PMMA photonic crystal hydrogels upon adding different amounts of water: 0, 25, 50, 75, 100, 125, 150 μL (by volume), respectively. The color changed from violet to blue, green, green, yellow, orange and red, respectively.

The resulting PC hydrogel has a violet color under light, with a narrow PBG, peaking at 460 nm (Fig. 2b, black curve). Because each NP was encapsulated with a layer of aqueous solution, the PC was able to respond to the water stimulation. As shown in the inset of Fig. 2b, the PC exhibited a colour change from violet to blue, green, yellow, orange and finally to red, upon increasing the volume of water from 0 to 150 μL. Fig. 2b demonstrates the reflection spectra that reveal the PBGs, positioned at 460, 484, 502, 543, 580, 613, and 618 nm, respectively. The color appearance of the PC can be described using Bragg's law (eqn (1)), where *λ* is the wavelength of the reflected light, *d* is the PC lattice space, and *θ* is the angle between the incident light and diffracting plane.^18^ When *θ* is fixed, *d* is a key factor that determines the PBG (*i.e.*, *λ*) of the PC. Usually, the NP size plays an important role in tuning *d*. For example, in a traditional copolymer PC system, the addition of water enlarges the NP size by swelling the hydrophilic block, resulting in an increase in the PC lattice space.8*a* In our system, however, the hydrophobic NFTPE/PMMA NPs were surrounded by SDBS aqueous solution; thus, it is believed that the water only tuned the aqueous medium which expands the PC lattice space (Fig. 2a), and consequently the PBG is bathochromically shifted. Moreover, the structure color of the swelled NFTPE/PMMA PC can be returned to violet after centrifugation (Fig. S2†). This is because the PC lattice space was reduced by extracting the water.

1 *λ* = 2*d* sin *θ*

**Fig. 3:**
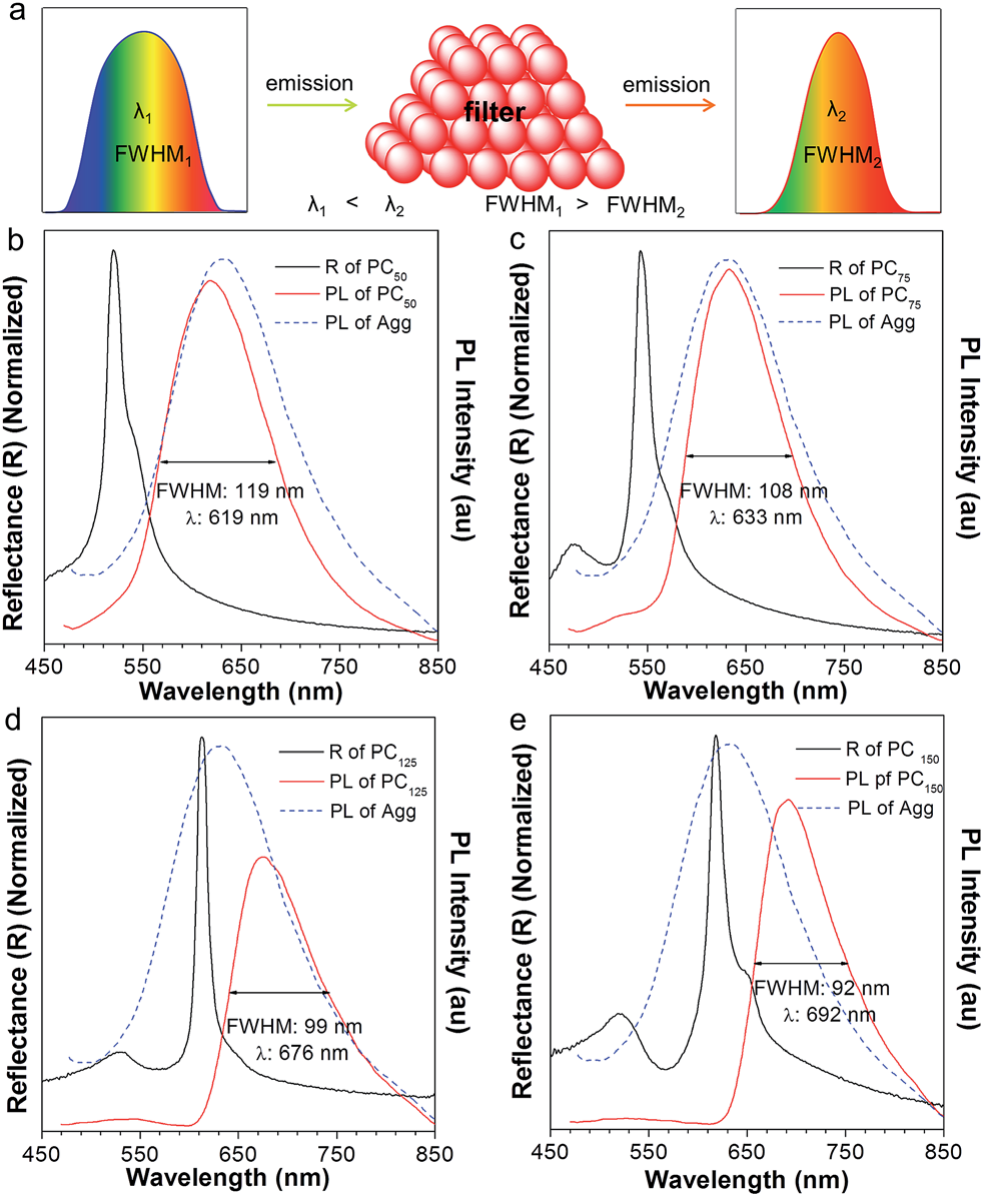
(a) Schematic illustrating the filter effect of the luminescent photonic crystal, where the emission peak red shifts and the FWHM is narrowed. (b–e) The fluorescence spectra of AIE1 NFTPE (blue) dissolved in THF : H_2_O = 1 : 9 solution, complemented with the fluorescence (red) and reflection spectra (black) of the NFTPE/PMMA photonic crystal with different amounts of added water: (b) 50 μL, (c) 75 μL, (d) 125 μL, and (e) 150 μL.

To our surprise, the luminescent PC has unique emission properties under the stimulation of water. It works as a filter (Fig. 3a), promoting the emission peak of NFTPE to red-shift from 583 to 692 nm, and narrowing the FWHM from 137 to 92 nm (Fig. 3b–e). As discussed above, such a water-active stimulus resulted in the red-shift of the PBG (Fig. 2b). Thus, we speculate that the PBG of the PC should play a critical role in tuning the AIEgen emission. To understand the effects of the PBG-induced shift, we must carefully check the relationship between the PBGs and the emission spectra under different conditions.

We chose the emission of the NFTPE aggregates formed in a THF/water = 1 : 9 solution as a reference, which emitted at 630 nm (Fig. 3b–e and S1,† blue curve). Before adding water, the emission of the NFTPE/PMMA PC is located at 583 nm, which is blue-shifted compared to that of the pure NFTPE aggregates (Fig. S1a†). This may be ascribed to the twisted intramolecular charge transfer (TICT) characteristic of NFTPE.^19^ It is well known that such TICT is sensitive to variation in the solvent polarity. Thus, the NFTPE emission is blue-shifted from a polar solution (*i.e.*, THF/water = 1 : 9 mixture) to a weak polar one (*i.e.*, PMMA). As shown in Fig. S1a,† the PBG of the PC hydrogel (460 nm, black curve) is at the left of the emission of NFTPE without adding water, far from the emission of AIE1 with minimum overlap. In this case, the PBG shows little effect on the emission shift. On increasing the water content, it is found that the PBG plays an increasingly prominent role in tuning the emission of NFTPE. At a low water content (*V*_adding water_ = 25 μL), the PBG slightly overlapped with the blue band-edge of the emission, inducing the NFTPE emission at 611 nm (Fig. S1b†). When the water volume was increased from 25 to 75 μL, the PBG was further red-shifted which enhanced its intimate contact with the emission spectra (Fig. 3b and c). The PBG has the ability to inhibit the propagation of photons if their energy is inside the PBG. That is, the emission is quenched in the wavelength range of the PBG.^12^,^20^ For example, in Fig. 3b and c, the emissions around 502 and 543 nm were suppressed. This may be due to (a) the forbidden effect of the PBG which impedes the emitted light from being detected, and (b) the energy transfer where the suppressed energy in the blue-wavelength region (*i.e.*, inside the PBG) is transferred to the low energy region. Therefore, the emission was bathochromically shifted from 611 to 633 nm, accompanied by the shrinking of the FWHM from 127 to 108 nm (Fig. 3b and c). On further increasing the water content to 125 and 150 μL, the PBGs completely overlapped with the emission spectra, resulting in the emissions below 613 and 618 nm being fully suppressed (Fig. 3d and e). This was due to the intramolecular energy transfer, and the NFTPE/PMMA PC emitted red light at 676 and 692 nm, with the FWHM reduced to 99 and 92 nm, respectively. The PBG-controlled emission allows the selective choice of an arbitrary light from just one material. Importantly, such a PBG-induced filter effect might be capable of modulating the broad emissions of AIEgens, benefiting their optoelectronic and biological applications. It is well known some AIEgen materials can change their colors and emission bands due to the transition of the crystalline structure or molecular packing induced by mechanical force or heating. However, in our system, the PBG-tuned emission change breaks such a traditional mode, and might open a new door to realize the emission modulation of all AIEgens, not only limited to materials with mechanochromic properties.

**Fig. 4:**
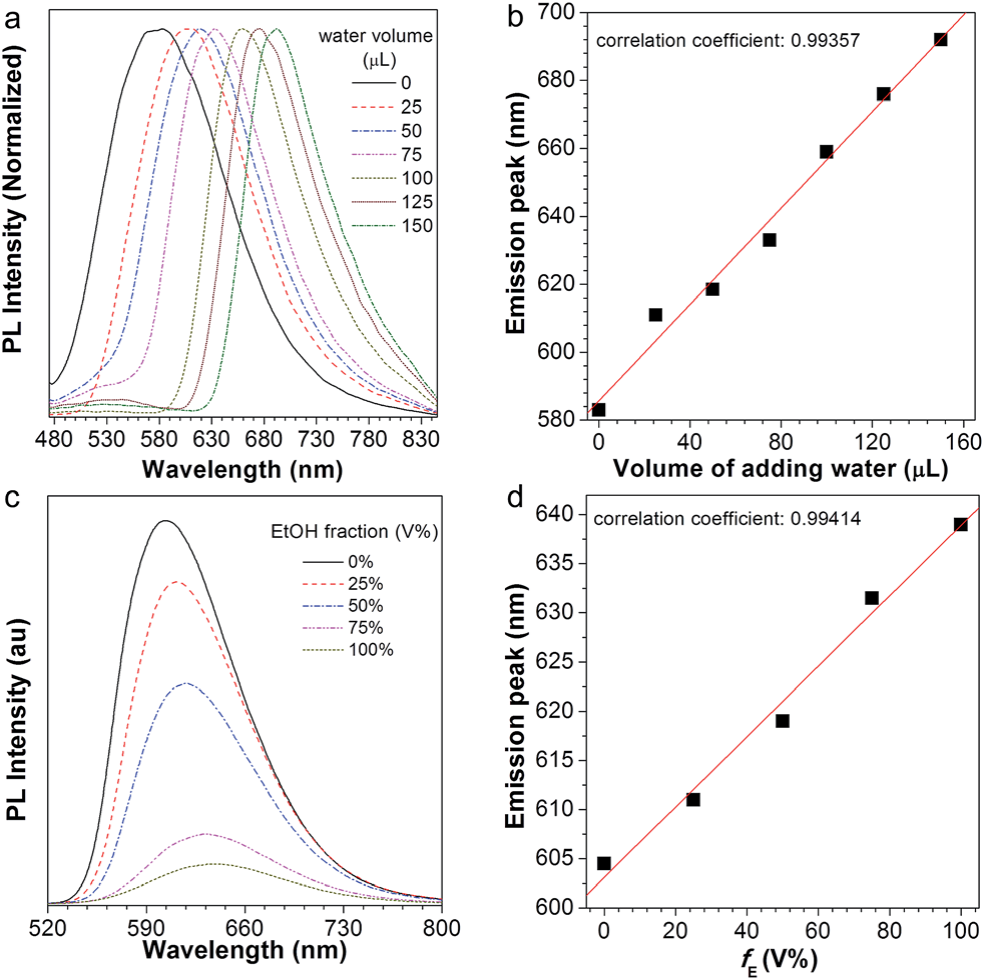
(a) Normalized PL spectra of the NFTPE/PMMA photonic crystal with different added amounts of water: 0, 25, 50, 75, 100, 125, and 150 μL (by volume), respectively; (b) linear relationship between the emission peak and the volume of added water. (c) PL spectra of the NFTPE/PMMA photonic crystal with an added ethanol/water mixture with different ethanol amounts: 0, 25%, 50%, 75%, and 100% (volume fraction, *V*%), respectively; (d) linear relationship between the emission peak and the amount of added ethanol (*f*_E_).

After normalization, it is clear that the emission peak of AIE1/PMMA PC exhibits a red shift in response to the increase in water level (Fig. 4a). Furthermore, a linear relationship between the emission peak *versus* the volume of added water was identified, as shown in Fig. 4b, which allows the quantitative detection of humidity. Such PBG-controlled emission shift is attributed to water permeation leading to the swelling of the aqueous medium, which could be regarded as an external stimuli-tuning process.

Since organic solvents can influence the mobility of PMMA NPs, we studied the influence of ethanol on the emission of the NFTPE/PMMA PC. In Fig. 4c, the emission of the luminescent PC was red-shifted when an ethanol/water mixture (25 μL) containing variable ethanol contents (*V*% = 0, 25%, 50%, 75%, and 100%, respectively) was added. At a low ethanol level (*V*% = 0), only the water-active stimuli dominates. With increasing ethanol content, the emission red-shift results from two factors: one is the swelling of the aqueous medium induced by the water–ethanol synergy (defined as external tuning); the other is the expansion of the luminescent NPs conducted by the ethanol (defined as internal tuning). Although such external-internal synergy-active tuning is gentle (red-shift of 34 nm), a linear relationship between the emission peak and the volume of added ethanol can also be achieved (Fig. 4d). Thus, the moderate and double tuning could be applied to detect the alcohol in wine. In contrast to the water-active stimuli, in cases where ethanol participated in the tuning, the emission intensities were sharply reduced, especially at a high ethanol level (Fig. 4c). We postulated that this was due to two possibilities: (a) the PC structure might be partially destroyed by ethanol; (b) the aggregated and rigid AIEgen NFTPE might become loose due to the swelling of ethanol, and thus the molecular motion of NFTPE is active, giving rise to a decrease in emission intensity.

With this new understanding, another AIEgen TPE-containing diacrylate (AIE2, Fig. S3a†) was employed to study the PBG-controlled emission shifting. Because of two active C=C double bonds, AIE2 could chemically crosslink with MMA during the emulsion polymerization, forming luminescent polymer NPs.16*a* This behavior is different from AIE1 NFTPE which is only physically incorporated into the PMMA NPs. However, after centrifugation at high speed, the resulting AIE2/PMMA PC demonstrated a similar phenomenon to NFTEP/PMMA upon stimulation with water. As shown in Fig. S3b†, the reflection peak displayed a red shift from 411 to 450, 482, 529, 563, 583 and 600 nm, when the water volume was progressively increased from 0 to 25, 50, 100, 125, 150, and 175 μL, respectively. Consequently, the AIE2/PMMA PC exhibited bright structure colors, varying from violet, to blue, sky blue, green, yellow-green, yellow, and to red (Fig. S3b,† inset).

**Fig. 5:**
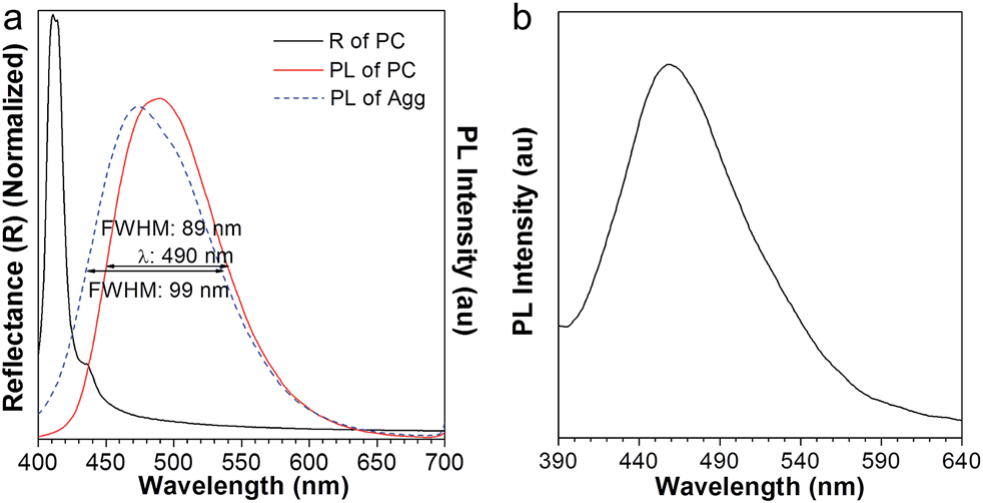
(a) The PL spectra of the AIE2 aggregates (blue) formed in THF : H_2_O = 1 : 9 solution, complemented with the fluorescence (red) and reflection spectra (black) of the AIE2/PMMA photonic crystal before adding water. (b) The PL spectra of AIE2/PMMA nanoparticles after destroying the photonic crystal structure.

**Fig. 6:**
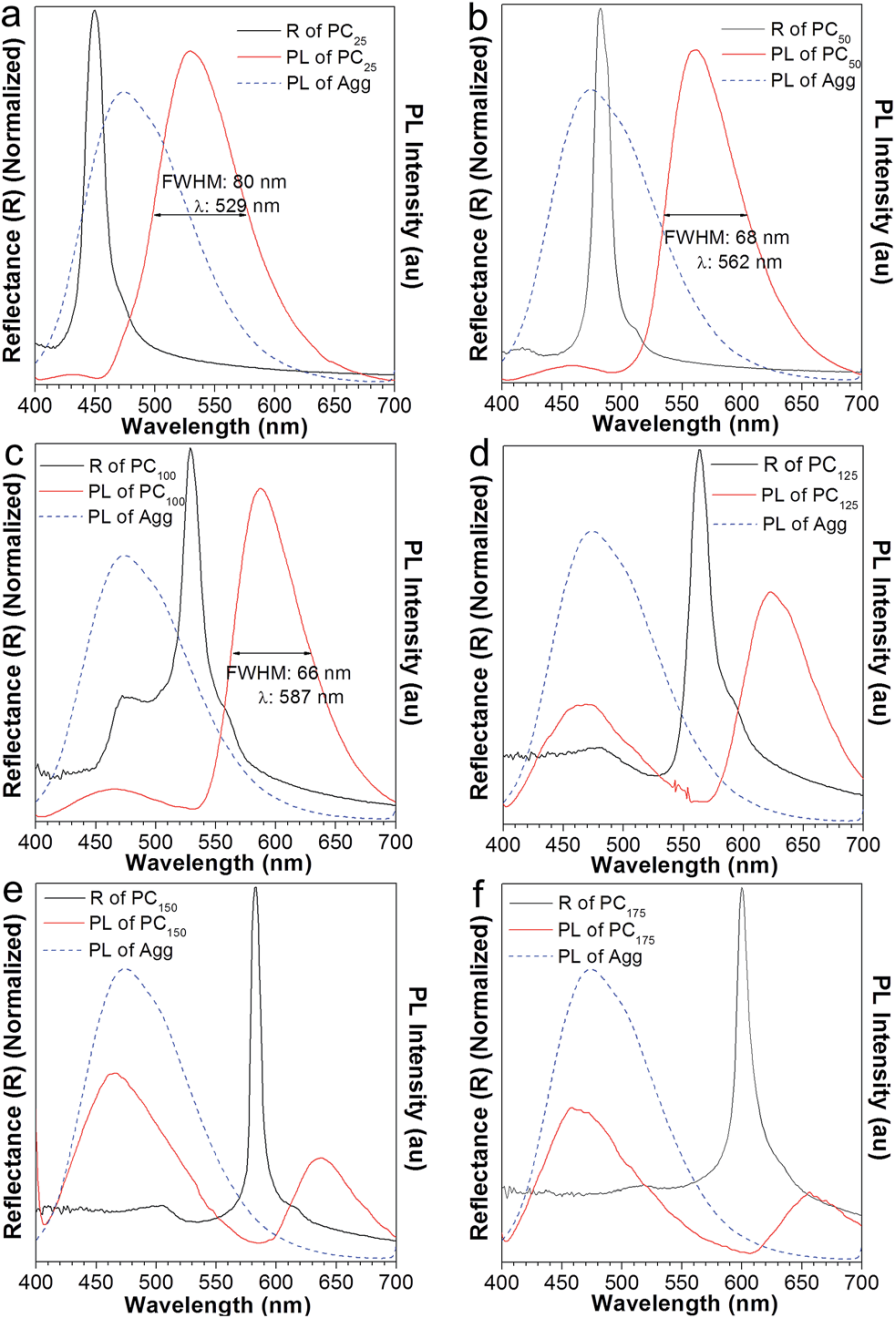
The PL spectra of AIE2 aggregates (blue) formed in THF : H_2_O = 1 : 9 solution, complemented with the fluorescence (red) and reflection spectra (black) of the AIE2/PMMA photonic crystal with the addition of different volumes of water: (a) 25 μL, (b) 50 μL, (c) 100 μL, (d) 125 μL, (e) 150 μL, and (f) 175 μL.

The aggregates of AIE2 assembled in a THF/water = 1 : 9 solution emitted blue light, peaking at 470 nm (Fig. 5a, blue curve). However, in the absence of added water, the obtained AIE2/PMMA PC emitted at 490 nm (Fig. 5a, red curve), showing a red-shift compared to the pure AIE2 aggregates. In this case, the PBG-induced emission shift has come into play. In Fig. 6a, the reflection spectrum of AIE2/PMMA PC overlapped with the blue band-edge of its emission, thus resulting in the light inside the PBG (*i.e.*, below 430 nm) being suppressed. On the principle of intramolecular energy transfer, the emission is bathochromically shifted with a decrease in the FWHM from 99 to 88 nm. If destroying the PC structure using a large amount of water, the emission of AIE2/PMMA NPs was recovered to 460 nm (Fig. 5b). This indicates that AIE2 was polymerized with MMA through covalent bonds and uniformly distributed in the PC, and that the synthesized polymer aggregates were more sensitive to the variation in the surrounding environment due to their higher hydrophobicity.16*a*

To further investigate the effect of the PBG in modulating the emission of AIE2, we added different volumes of water into the AIE2/PMMA PC. With the increase in water content from 25 to 75 and 100 μL, the PBG band (black curve in Fig. 6) shifted from high energy to the middle and then to the low energy region of the emission spectra. As a result, the AIE2 emission peak moved to long wavelengths from 529, to 562 and 587 nm, respectively, accompanied by a lessening of the FWHM from 80 to 68 and 66 nm (Fig. 6a–c). Such phenomena are consistent with those occurring in the NFTEP/PMMA PC system (Fig. 3). Thus, it is further proved that the PBG plays a dominant role in tuning the AIE emission.

On increasing the volume of added water to 125 μL, an unconventional phenomenon was observed, where the emission of the AIE2/PMMA PC was red-shifted to 622 nm, and simultaneously a new peak appeared at 470 nm with a relatively low emission intensity (Fig. 6d). On adding more water into the PC system, the AIE2 emission still split into two peaks. In Fig. 6e and f, for example, the emission was further shifted to 637 and 656 nm, and new emissions were observed at 466 and 460 nm, respectively. However, in these two cases, the intensities of the new emission peaks became stronger than those of the shifted ones. This observation was in contrast to the NFTPE/PMMA PC system, which only produced an emission red-shift in response to the water stimulation. We realized that such an unconventional phenomenon is dependent on the optical properties of the AIEgens themselves. For AIE1 NFTPE emission, the energy population is concentrated at 500–750 nm, and the PBG is always on the left side of its emission spectrum (Fig. 3), so that the light with little energy population is blocked because of the PBG effect, promoting the red-shift of emission based on the intramolecular energy transfer. However, AIE2 exhibits blue emission, for which the energy population lies at a short wavelength (*i.e.*, 400–580 nm). Thus, when the PBG shifted to the right side of the AIE2 emission spectrum, such as 563 nm or even further, it displayed a weak role in suppressing the blue emission, giving rise to the re-emergence of the characteristic peak of AIE2. On the other hand, the emission inside the PBG wavelength was inhibited, resulting in the splitting of the emission spectrum, and the red-shift of emission due to the intramolecular energy transfer. Compared to the blue emission, the relative emission intensity of the shifted peak was gradually reduced. This is ascribed to the synergy of the following factors: (a) the red light has no contribution to the emission of AIE2; (b) the PBG suppresses partial red emission; and (c) little energy is transferred. Such a unique phenomenon provides an opportunity to gain two color emissions from a single material by combining the PC properties and AIEgen photophysical properties. Therefore, it is believed that such a luminescent PC as a good intramolecular filter would be able to create a designed multiplex light.

**Fig. 7:**
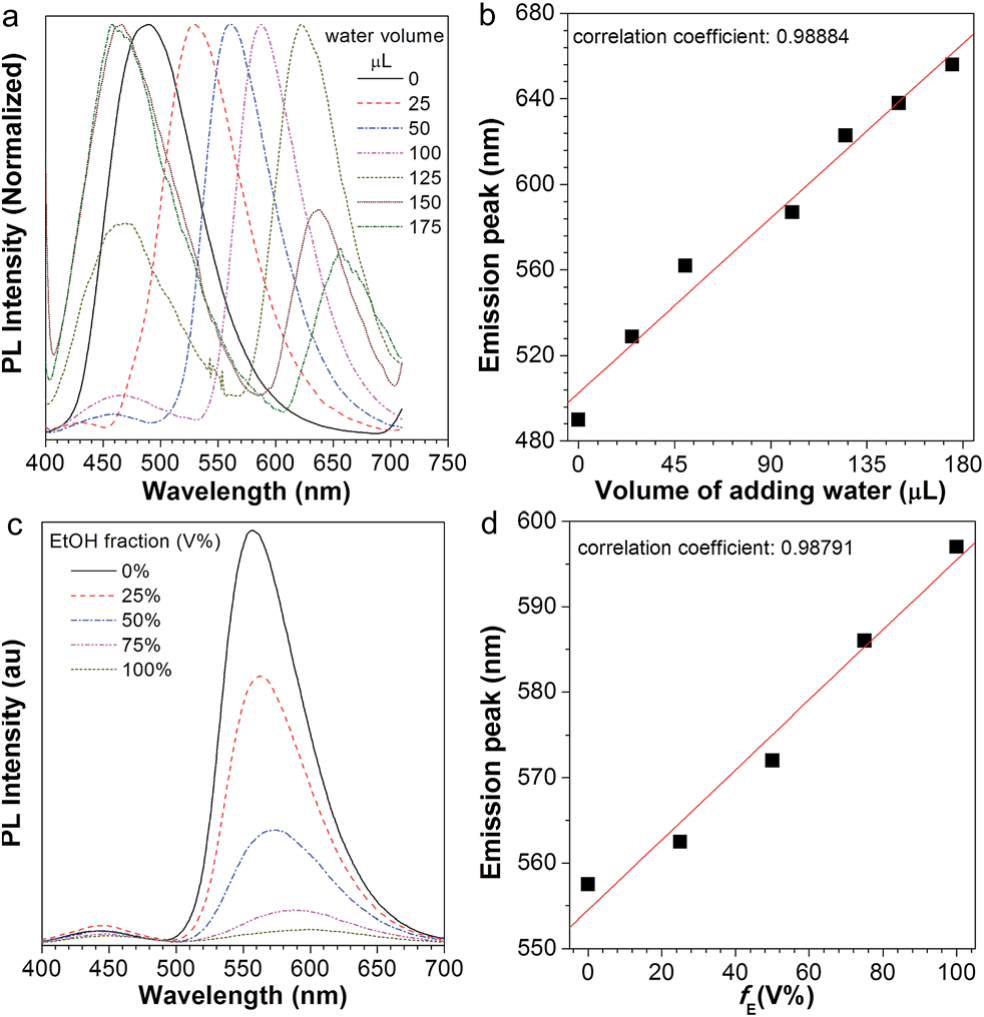
(a) Normalized PL spectra of the AIE2/PMMA photonic crystal on adding different amounts of water: 0, 25, 50, 100, 125, 150, and 175 μL (by volume), respectively; (b) linear relationship between the emission peak and the volume of added water. (c) PL spectra of the AIE2/PMMA photonic crystal with an added ethanol/water mixture with different ethanol amounts: 0, 25%, 50%, 75%, and 100% (volume fraction, *V*%), respectively; (d) linear relationship between the emission peak and the amount of added ethanol (*f*_E_).

In the presence of the water-active stimuli, the emission change of AIE2/PMMA PC is evidently reflected in Fig. 7a. The emission peak and the water volume perfectly conform to a linear relationship (Fig. 7b). We also found that the AIE2/PMMA PC was capable of tuning its emission intensity and wavelength in response to the variation in ethanol, and a linear relationship was obtained (Fig. 7c and d). These results indicate that the AIE2/PMMA PC possesses dual-functionality to quantitatively test humidity and alcohol. Although different AIEgens were used, the similarity in the tunability and multi-function response is a strong indication for the generality of our AIE/PMMA PC system.

## Conclusions

In this study, we exploited a simple method, emulsion polymerization combined with centrifugation, for the generic fabrication of luminescent photonic crystals from AIEgens. The resulting AIE/PMMA photonic crystal exhibits a prominent water-responsive optical property change, where a linear relationship was achieved. By carefully tuning the photonic crystal structure, its reflection and emission color were red-shifted at the same time. It was found that the ability of the PBG to selectively modulate and narrow emission allowed the luminescent PC to act as an intramolecular filter. This unique property thus gives insights into tuning an arbitrary emission color from only one material. Meanwhile, the choice of AIEgen is important, particularly for the modulation of two emission colors. Our understanding of the emission-change mechanism allowed the use of different solvents for the modulation. Further success has been achieved in the ethanol-conducted emission change.

The luminescent photonic crystal system, with multi-functionality and controllability, could be potentially useful as a humidity and alcohol sensor, as well as a photoelectric device component. Thus, the perfect encounter between AIEgens and photonic crystals will surely bring new opportunities to greatly exploit AIEgens in biological and optoelectronic applications.

## Supplementary Material

Supplementary information
