# Supplementary material for: Luminescent photonic crystals with multi-functionality and tunability
Source: Chem Sci. 2016 May 25;7(9):5692–8. doi: 10.1039/c6sc01703g (PMC6022079; doi:10.1039/c6sc01703g)
Supplement: Supplementary file 1 [file SC-007-C6SC01703G-s001.pdf]

Electronic Supplementary Material (ESI) for Chemical Science.
This journal is © The Royal Society of Chemistry 2016

-Supporting Information-

Luminescent Photonic Crystals with Multi-functionality and Tunability

Hong Wang,[ab‡] Xinggui Gu,[ab‡] Rongrong Hu,[c] Jacky W. Y. Lam,[ab] Deqing Zhang,[d] and Ben Zhong Tang[abc]*

a HKUST-Shenzhen Research Institute, No. 9 Yuexing 1st RD, South Area, Hi-tech Park, Nanshan, Shenzhen 518057, China

b Department of Chemistry, Hong Kong Branch of Chinese National Engineering Research Center for Tissue Restoration & Reconstruction, Institute for Advanced Study, Institute of Molecular Functional Materials, Division of Biomedical Engineering, Division of Life Science and State Key Laboratory of Molecular Neuroscience, The Hong Kong University of Science & Technology, Clear Water Bay, Kowloon, Hong Kong

c Guangdong Innovative Research Team, SCUT-HKUST Joint Research Laboratory, State Key Laboratory of Luminescent Materials and Devices, South China University of Technology, Guangzhou 510640, China

d Beijing National Laboratory for Molecular Sciences, Key Laboratory of Organic Solids, Institute of Chemistry, Chinese Academy of Sciences, Beijing 100190, PR China

Email: tangbenz@ust.hk

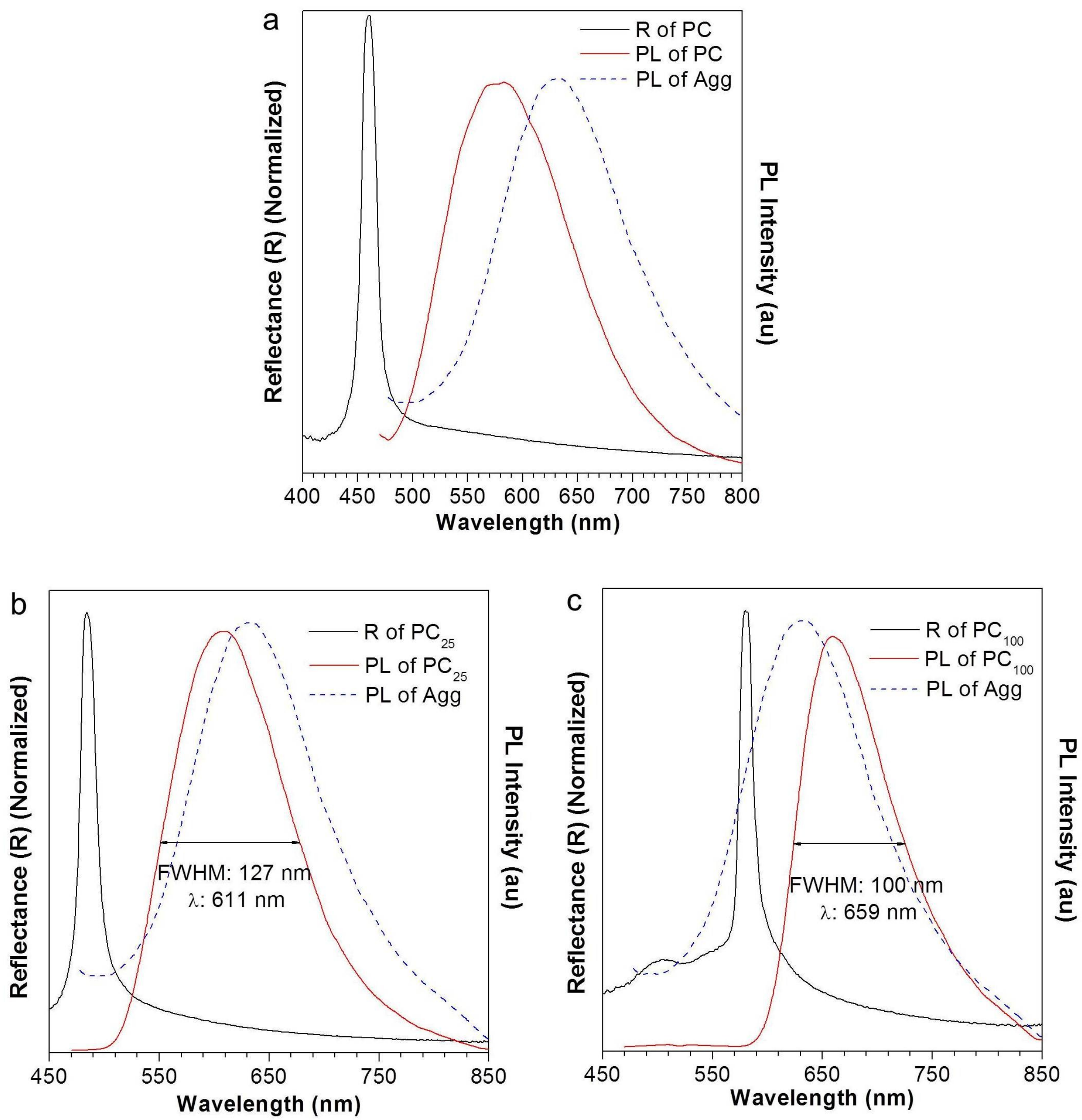

Figure S1. The fluorescence spectra of AIE1 NFTPE (blue) dissolved in THF : H_2O = 1:9 solution, complemented with the fluorescence (red) and reflection spectra (black) of the NFTPE/PMMA photonic crystal with adding different amounts of water: (a) 0 µL, (b) 25 µL, and (c) 100 µL, respectively.

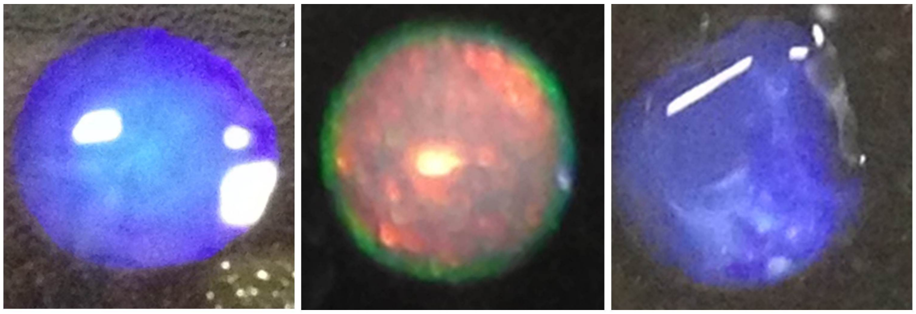

before adding water after adding water after centrifugation

Figure S2. Photographs of NFTEP/PMMA PC before adding water (left), after adding water (middle), and after centrifugation (right), respectively.

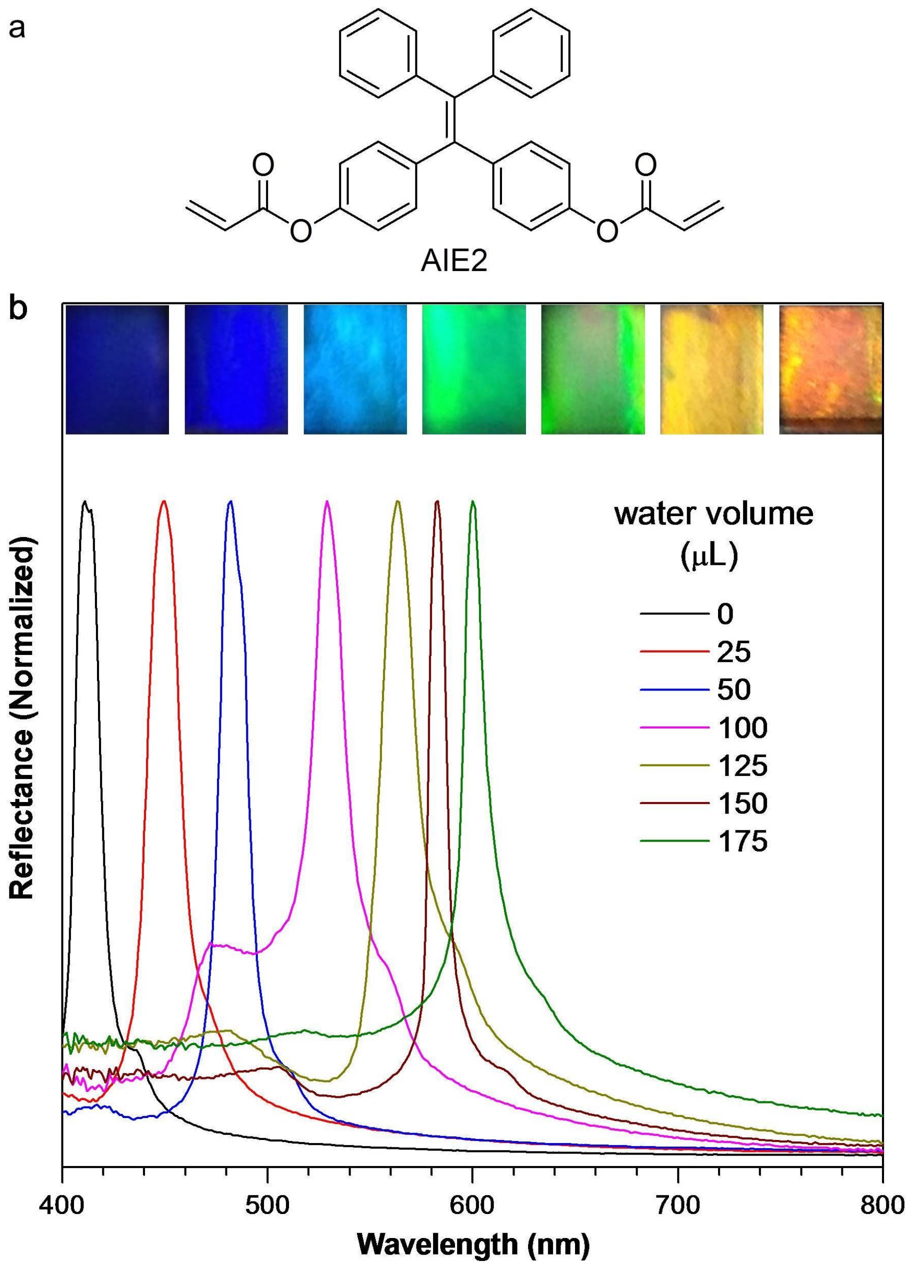

Figure S3. (a) Molecular structure of AIE2. (b) Photographs (inset) and corresponding reflection spectra of the AIE2/PMMA photonic crystal hydrogel with adding different amounts of water: 0, 25, 50, 100, 125, 150, and 175 μL (by volume), respectively. The color changed from violet, blue, sky blue, green, yellow-green, yellow, to red, respectively.
